# Hydrostatic pressure in combination with topographical cues affects the fate of bone marrow‐derived human mesenchymal stem cells for bone tissue regeneration

**DOI:** 10.1002/jbm.a.36267

**Published:** 2017-10-23

**Authors:** Yvonne Reinwald, Alicia J. El Haj

**Affiliations:** ^1^ Institute of Science and Technology in Medicine, Keele University, Medical School, Guy Hilton Research Centre, UHNM Stoke‐on‐Trent United Kingdom; ^2^ Department of Engineering, School of Science and Technology Nottingham Trent University Nottingham United Kingdom

**Keywords:** mechanical stimulation, hydrostatic pressure, stem cells, nano‐topography, bone regeneration

## Abstract

Topographical and mechanical cues are vital for cell fate, tissue development *in vivo*, and to mimic the native cell growth environment *in vitro*. To date, the combinatory effect of mechanical and topographical cues as not been thoroughly investigated. This study investigates the effect of PCL nanofiber alignment and hydrostatic pressure on stem cell differentiation for bone tissue regeneration. Bone marrow‐derived human mesenchymal stem cells were seeded onto standard tissue culture plastic and electrospun random and aligned nanofibers. These substrates were either cultured statically or subjected to intermittent hydrostatic pressure at 270 kPa, 1 Hz for 60 min daily over 21 days in osteogenic medium. Data revealed higher cell metabolic activities for all mechanically stimulated cell culture formats compared with non‐stimulated controls; and random fibers compared with aligned fibers. Fiber orientation influenced cell morphology and patterns of calcium deposition. Significant up‐regulation of Collagen‐I, ALP, and Runx‐2 were observed for random and aligned fibers following mechanical stimulation; highest levels of osteogenic markers were expressed when hydrostatic pressure was applied to random fibers. These results indicate that fiber alignment and hydrostatic pressure direct stem cell fate and are important stimulus for tissue regeneration. © 2017 The Authors Journal of Biomedical Materials Research Part A Published by Wiley Periodicals, Inc. J Biomed Mater Res Part A: A: 629–640, 2018.

## INTRODUCTION

Anchorage‐dependent cells are able to recognize their environment and react to its properties when adhering onto biomaterial substrates through integrin‐mediated focal adhesion.^1^ These cell‐substrate interactions trigger cell signaling cascades and influence stem cell fate. Stem cell fate is further controlled through substrate topography, such as surface roughness of the underlying surface and isotropic or anisotropic surface patterns, but also three‐dimensional microenvironment, biochemical signals and external forces.^2^, ^3^, ^4^ Traditionally, cells are cultured 2D *in vitro*. However, differences in cell adhesion, migration, proliferation, and gene expression have been observed when comparing 2D with 3D culture.^5^ Hence, the focus has been on the development of 3D artificial matrixes that aim to mimic the extra cellular matrix (ECM), the native surrounding of cells, which is formed of pores, ridges, and nanofibers.^6^, ^7^ Those scaffolds are required to provide cell support, to facilitate cell proliferation, differentiation and to offer topographical guidance.^8^, ^9^ Due to its cost‐effectiveness and versatility electrospinning has been utilized for the fabrication of fibrous scaffolds with diameters between nanometers to several micrometers.^6^ Commonly used materials for electrospinning are poly (ɛ‐caprolactone) (PCL) and PCL‐composites, which are attractive for bone tissue regeneration due to their mechanical properties, slow degradation rate, low cost and lack of toxicity.^10^, ^11^, ^12^ Due to their dimension and fibrous structure electrospun nanofibers resemble the native bone matrix and are thought to be an ideal artificial matrix to provide cues for the formation of calcified bone tissue.^13^, ^14^ Several groups have investigated the suitability of electrospun nano‐and microfibers for bone formation.^10^^–^ 12

Random and aligned PCL fibers have been shown to affect cell spreading and elongation; and hence matrix production and calcification.^15^, ^16^ Aligned fibers are known to guide cells along fiber direction, whereas cells cultured on random fibers maintain a rounded shape.^17^, ^18^

The alignment of collagen is of special importance for hard tissues such as bone since the mechanical strength of the calcified matrix depends on the alignment of collagen fibers in the native tissue.^19^ It was reported that collagen fibers extended from all directions of polygonal cells on random nanofibers, whereas collagen on aligned fibers was oriented along the polymeric fibers indicating that collagen fiber orientation and matrix deposition followed the alignment of polymeric fibers. In conclusion, fiber alignment also influences matrix deposition and mechanical properties of the artificial matrixes.^20^, ^21^

Fiber diameter is another important property of electrospun scaffolds, which influences cell response. Bone marrow stromal cells and osteoblast‐like cells exhibited higher proliferation and differentiation as well as an increased alkaline phosphatase (ALP) production on sub‐micrometer sized fibers compared with micrometer‐scaled fiber meshes.^10^, ^11^, ^16^

Fiber membranes promote mesenchymal stem cell (MSC) adhesion and proliferation. Another study by Shin et al. (2004)^12^ investigated the culture of bone marrow derived rat MSC on random PCL for 4 weeks in a rotary bioreactor system before implanting these *in vivo* for a further 4 weeks. Results showed that electrospun fiber meshes facilitated MSC differentiation into osteoblasts, collagen type‐I production and mineralization. Tuzlakoglu et al. (2005)^22^ described the fabrication of a fibrous scaffold by depositing PCL/starch nanofibers onto PCL/starch microfibers to resemble the biophysical structure of ECM. These random nanofibers enabled osteoblast‐like cells to bridge microfibers and were further found to increase the cell metabolic activity.

For the generation of functional tissues it is important to replicate the structural and mechanical environment cells experience *in vivo*. Mechanical forces have been recognized to play a key role for stem cell fate decisions. However, it is challenging to examine biomechanical mechanisms *in vivo*. To investigate cell fate and tissue maturation when external forces are applied and to provide physical growth environments for cells and tissues *in vitro* bioreactors have been developed, subjecting cells to various types of mechanical forces, such as hydrostatic pressure.^23^, ^24^ Hydrostatic pressure is present in the body in various magnitudes and has been shown to be a relevant physical stimulus for load‐bearing bones where osteocytes in the canaliculi lacuna network are exposed to a hydrostatic pressure of around 270 kPa.^25^, ^26^ Furthermore, hydrostatic pressure is also important for many biological systems for transducing forces in fluid‐filled tissues. During movement compressive loading and unloading of hydrated tissues result in changes of the interstitial fluid pressure.^26^ Hydrostatic pressure has further been shown to enhance mineralization and bone formation when applied to osteoblast‐like cells, cell‐seeded constructs, and *ex‐vivo* organotypically cultured chick femurs.^25^, ^26^, ^27^

To the authors knowledge the combinatory effect of topography and hydrostatic pressure on human bone marrow‐derived mesenchymal stem cells (hBMSC) for bone tissue engineering application has not been studied previously. This study aims to provide a better understanding of how mechanical stimulation in synergy with PCL fiber alignment affect the osteogenic potential of hBMSC and hence their regenerative potential for bone tissue engineering. Therefore, hBMSC were cultured onto random and aligned electrospun PCL fibers over 21 days. Samples were subjected to an intermittent hydrostatic pressure (IHP) stimulation regime at 270 kPa, which replicates the pressure within the canaliculi lacuna network of load‐bearing bones and 1 Hz, the human pulse‐rate. These conditions resemble physiological relevant mechanical stimuli.

## MATERIALS AND METHODS

### Cell culture and mechanical stimulation

**Figure 1:**
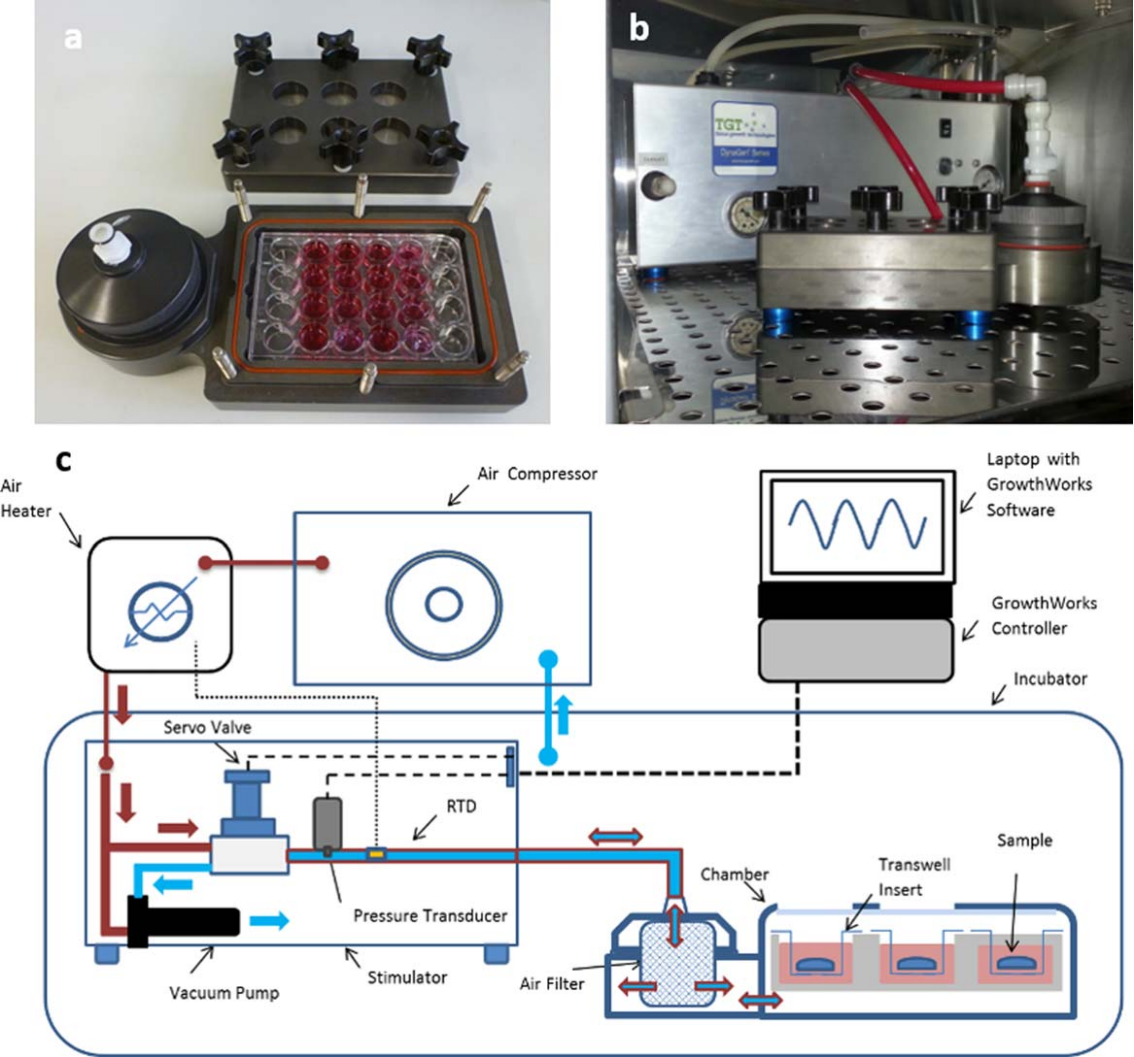
Hydrostatic pressure bioreactor. The bioreactor chamber (a) fits standard tissue culture well plates and is placed in the (b) cell culture incubator after loading. (c) The schematic shows the complete bioreactor system. The bioreactor enables either cyclic or continuous mechanical stimulation of cells and tissue engineered constructs at pressures ranging between 0 and 270 kPa at 0–2 Hz frequency. The figure was adapted from Reinwald et al. (2015).^25^

hBMSC were obtained from Lonza as bone marrow aspirate, isolated following the companies protocol and cultured in Dulbecco's Modified Eagle Medium (DMEM). DMEM was supplemented with 2 m*M* L‐glutamine, 10% fetal bovine serum, and 5× antibiotics/antimycotics (100 U/mL penicillin, 0.1 mg/mL streptomycin) and cultured at 37°C and 5% CO_2_ until confluent. Cells were trypsinised, counted and subsequently 2 × 10^4^ cells (passage 2) were seeded onto PCL fiber well plates, which were purchased from Nanofibre Solutions (USA) as 6‐well plate and 24‐well plate formats. Cell viability and morphological characterization of fibers were assessed in 6‐well plates. All other assays were performed in 24‐well plates. Cells were cultured using osteogenic medium (DMEM supplemented with 100 U/mL penicillin, 0.1 mg/mL streptomycin, 100 n*M* dexamethasone, 0.05 m*M* L‐ascorbic acid 2‐phosphate, 10 m*M* β‐glycerolphosphate). All reagents were purchased from Lonza unless otherwise stated. A schematic of the bioreactor is shown in Figure 1. Sample groups are described as random (R) and aligned (A) fibers and tissue culture plastic (TCP), which were either mechanically stimulated with IHP or statically cultured as control samples (C) over 21 days. Mechanical stimulation was performed in a custom designed hydrostatic pressure bioreactor (TGT/Instron, USA) at 270 kPa and 1 Hz for 60 min 5 days per week.

### Fiber morphology and chemical characterization of PCL fibers

Fibers were mounted on scanning electron microscopy (SEM) stubs (Philips 12 mm Aluminum stubs, AGAR Scientific LTD, UK) and gold sputter‐coated (Balzers SCD 030 sputtering device, Balzers Union Ltd, UK) for 4 min. Images were taken using a variable pressure SEM (Jeol JSM‐6060LV, Jeol LTD, UK). To visualize cells under SEM, scaffolds were osmium tetroxide stained before sputter‐gold coating. To determine fiber size and fiber size distribution, a minimum of hundred fiber diameters were measured using SEM accompanying software. In addition, chemical characterization of PCL nanofibers was performed in triplicate by Energy‐dispersive X‐ray spectroscopy (EDX) analysis using Hitachi TM3000 Scanning Electron Microscope and Quantax 70 software (Bruker).

### Cell metabolic activity and cell viability

To investigate cell metabolic activity samples were stained with MTT 3‐(4,5‐dimethylthiazol‐2‐yl)‐2,5‐diphenyl tetrazolium bromide reagent. Briefly, MTT reagent (Sigma, UK) was dissolved in phosphate buffered saline (PBS) to prepare a MTT stock solution (5 mg/mL). A one tenth dilution of MTT stock solution and serum‐free DMEM was added to the samples. Fibers were incubated for 3 h at 37°C and 5% CO_2_ following which the staining solution was aspirated and DMSO added to elute the formazan. After an incubation of 1 h at room temperature, the absorbance was read at 570 nm. MTT was performed on days 1 and 14. Three samples were prepared for each condition and each sample was read five times. hBMSC cell numbers were obtained from an MTT standard curve, which was prepared from hBMSC numbers ranging between 0 and 2 × 10^6^ cells (passage 2). Live/Dead stain was performed to assess cell viability on days 1, 7, and 14. The medium was aspirated and cells were washed with PBS. Live/Dead stain (Sigma, UK) was prepared following the suppliers instructions. Cells were incubated for 30 min at room temperature and then washed with PBS. Images were taken using a Nikon eclipse Ti imaging system.

### Calcium deposition

Calcium deposition was investigated using alizarin red stain. Therefore, fibrous scaffolds and TCP were fixed with 10% neutral buffered formalin at 4°C overnight. Samples were washed with PBS once and then stained for calcium deposition using 1% (w/v) aqueous Alizarin Red S (Sigma, UK) (pH 4.0), washed with dH_2_O twice and subsequently imaged using an EVOS light microscope. For semi‐quantification, the alizarin red stained area on each image (days 14 and 21) was determined in pixel using ImageJ. Three wells for each condition were imaged and three images were taken for each well. The field of view was chosen at random.

### Quantitative polymerase chain reaction

Quantitative polymerase chain reaction (qPCR) was performed in order to determine the expression of osteogenic and chondrogenic markers on days 1, 14, and 21. Briefly, RNA was isolated using Trizol reagent (Sigma, UK). RNA's purity and quantity was spectrophotometrically determined at 260 and 280 nm (Nanodrop). Subsequently, cDNA was synthesized using a High‐Capacity cDNA Reverse Transcription Kit (Life Technologies, UK). qPCR was performed on a light cycler (Agilent Technologies, UK) for ALP, SOX‐9, RUNX‐2, Collagen‐I (Col‐I), Osteopontin (OPN), and GAPDH. Primers and SYBR Green were obtained from Qiagen, UK. The expression values were calculated relative to GAPDH following the ΔCT method and fold increase was normalized to untreated control (non‐mechanically stimulated plates) (*n* = 3 samples and each sample was repeated three times).

### Statistical analysis

All experimental analysis was performed with *n* = 5 unless otherwise stated. Results are expressed as means ± SD and for gene expression as means ± SEM. Two‐way ANOVA and Tukey's tests for multiple comparisons were performed using Graph Pad Prism 6. A *p* values of < 0.05 was assumed as statistically significant.

## RESULTS

### Fiber morphology and size distribution of electrospun PCL fibers

**Figure 2:**
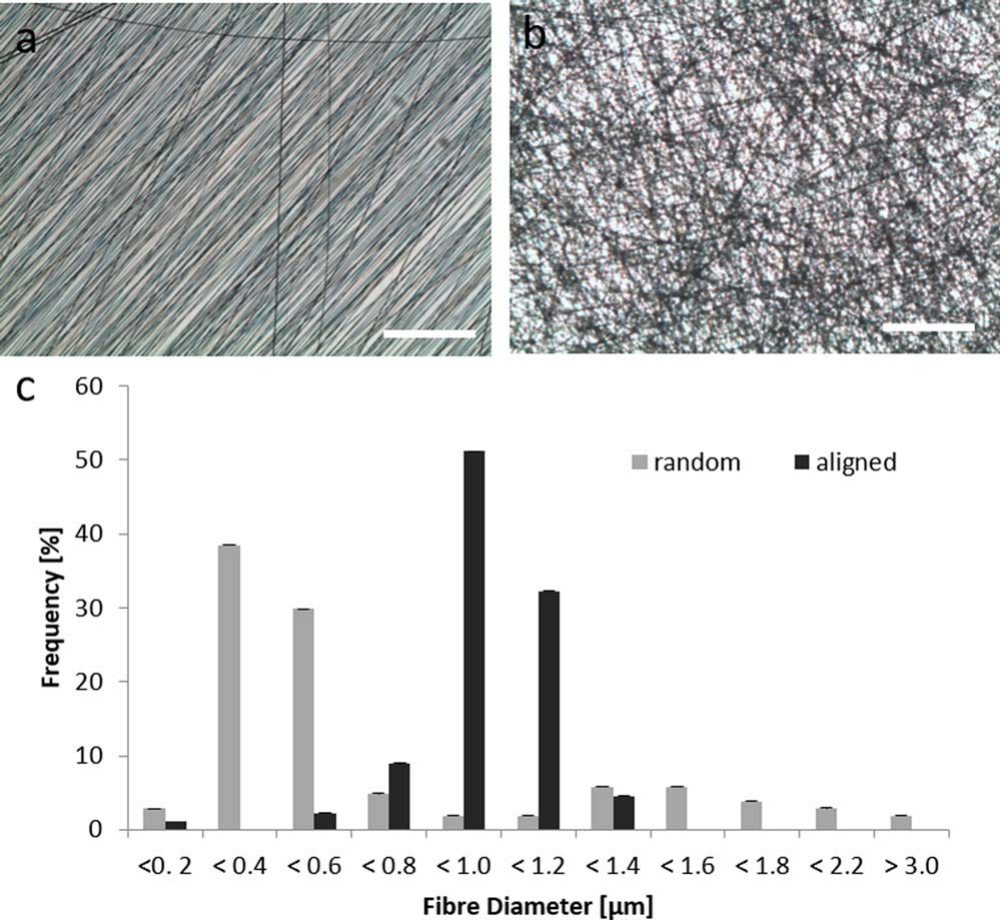
Electrospun fibers fabricated from PCL with random and aligned orientation. (a,b) Fiber orientation is shown on light microscopic images. (c) Fiber diameter distribution was determined from SEM images. Scale bar = 125 µm. SDs are shown as error bars (*n* > 90).

Fiber morphology of random and aligned fibers was investigated by light microscopy [Fig. 2(a,b)] and SEM [Fig. 2(c)]. Fiber size distributions ranged from 200 nm to 3.0 µm with >70% of fiber diameter below 800 nm (random fibers) and >80% below 1.2 µm (aligned fibers) (Fig. 2). As the histogram shows around 70% of random fibers are between 400 and 600 nm and around 80% of aligned fibers are between 1000 and 1200 nm. Overall, a more narrow size distribution was observed for aligned fibers. Average fiber diameters of 700 nm (random) and 940 nm (aligned fibers) were determined. The fiber sheet thickness was around 20 µm according to manufacturer's information.

### Cell metabolic activity and cell viability

**Figure 3:**
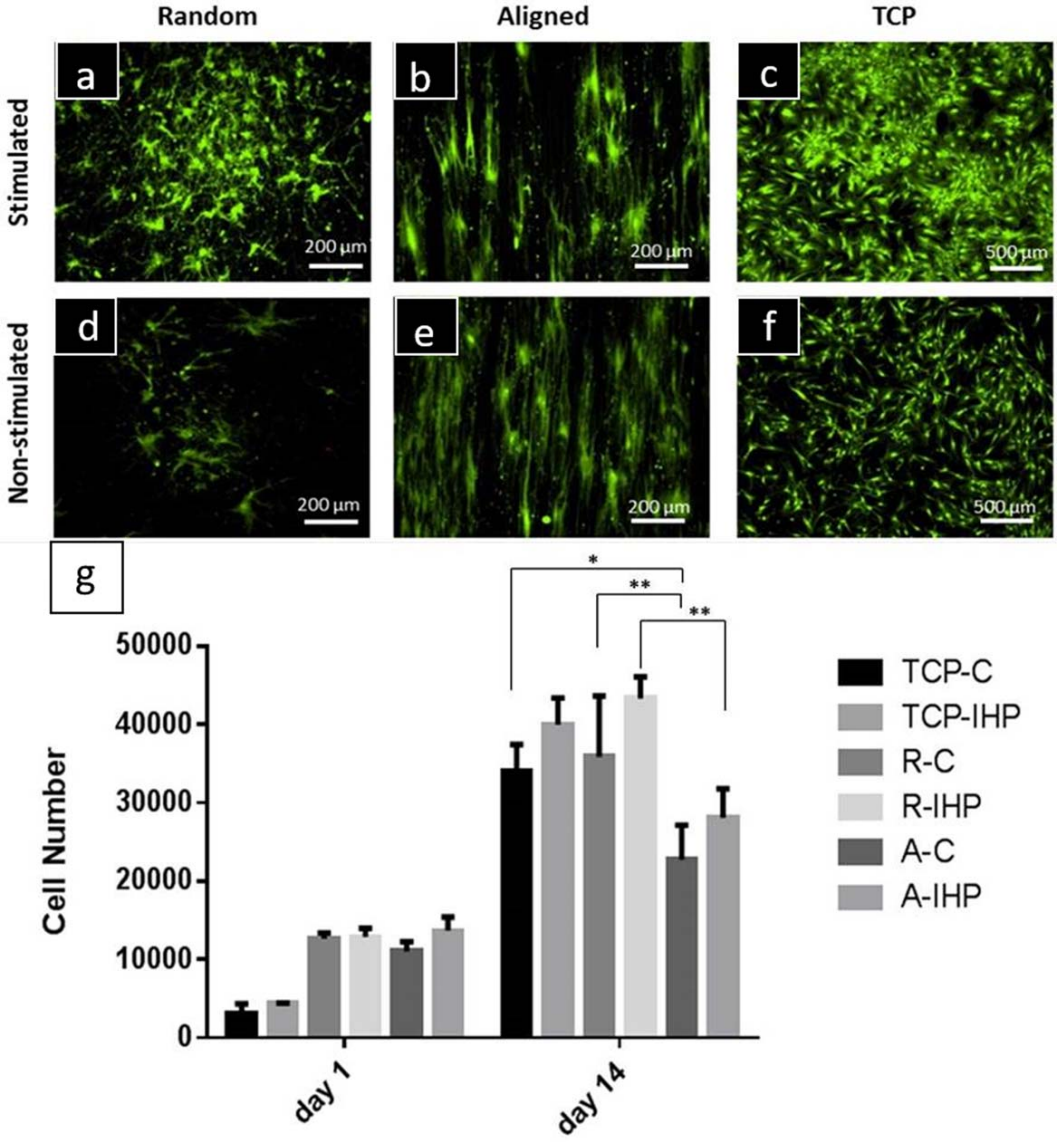
Viability and metabolic activity of hBMSC cultured on PCL nanofibers. a–f: Live/Dead stain revealed viable hBGMSC cultured on mechanical stimulated and non‐stimulated TCP, random and aligned fibers (day 14). g: Cell metabolic activity on random and aligned fibers and TCP was assessed by MTT. Tissue culture formats were subjected to hydrostatic pressure at 270 kPa, 1 Hz for 1 h and compared with non‐stimulated plates (C, non‐stimulated control; IHP, mechanically stimulated). SD is shown as error bars (*n* = 5).

Cells were viable on all cell culture formats [Fig. 3(a–f)]. Representative images for each group indicate enhanced cell proliferation under mechanical stimulation. In addition, cells cultured on TCP covered the bottom of the well plate homogenously [Fig. 3(e,f)], whereas hBMSCs cultured on aligned and random fibers oriented themselves according to fiber orientation [Fig. 3(a–d)]. Cells on aligned fibers were elongated and spindle‐shaped. Mostly rounded cells were observed on random‐oriented fibers.

For all tissue culture well formats metabolic activity increased supporting that the investigated cell cultured formats enhanced cell metabolic activity [Fig. 3(g)]. Results shown for day 1 indicate initial cell metabolic activities could result from differences in cell seeding numbers. However, cell numbers seeded on random fiber substrates were similar for R‐C and R‐IHP on day 1. After 14 days of mechanical stimulation cell metabolic activity was increased on R‐IHP (43,319 ± 2803) compared with R‐C (35,867 ± 7762) indicating a positive effect of hydrostatic pressure. Similar results were observed for TCP and aligned PCL fibers. Initial cell numbers were slightly lower on TCP‐C and A‐C compared with TCP‐IHP and A‐IHP. On day 14 metabolic activities of cells cultured on TCP‐IHP (39,982 ± 3427) and A‐IHP (28,043 ± 3780) were non‐significantly higher compared with TCP‐C (34,032 ± 3386) and A‐C (22,723 ±4419). However, TCP‐C was significantly increased compared with A‐C (*p* values = 0.0327). In addition, the metabolic activity on statically cultured random fibers (R‐C, 35,867 ±7762) was significantly higher than aligned fibers (A‐C, 22,723 ±4419) (*p* values = 0.0066) and R‐IHP (43,319 ± 2803) was significantly higher than A‐IHP (28,043 ± 3780, *p* values = 0.0010). Live/Dead stains in Figure 3 support these observations as more live cells were observed for random fibers and TCP compared with aligned fiber substrates. These results demonstrate that cell metabolic activity was enhanced on mechanically stimulated TCP and random fibers. Metabolic activities for cells cultured on random PCL fibers were higher than for aligned fibers, which suggested that random nanofibers promoted higher cell metabolic activity regardless of the applied mechanical stimulation regime.

### Chemical characterization of PCL fibers and calcium deposition

**Figure 4:**
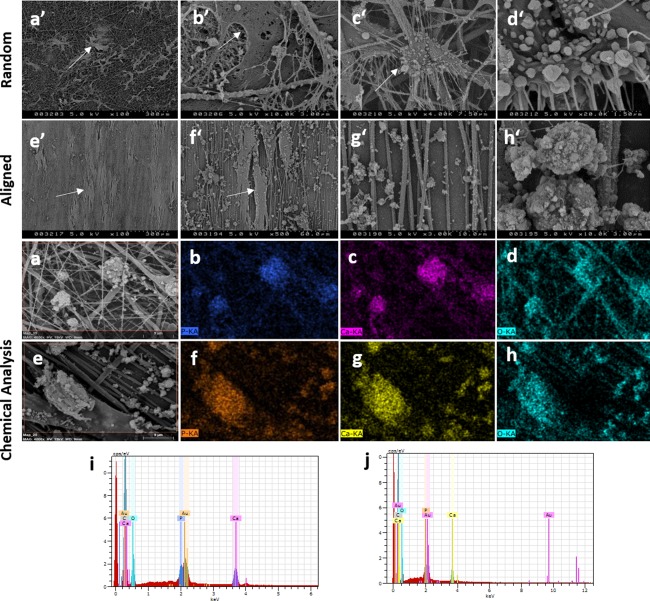
hBMSC orientation on PCL fibers and chemical characterization of mechanically stimulated nanofibers. After being cultured on fiber well plates for 21 days and subjected to cyclic hydrostatic pressure cells followed fiber orientation (cells are indicated by white arrows). a’–d’: SEM images were taken for random and (e’–h’) aligned fibers at (a’, e’) 100×, (f’) 500×, (g’) 3000×, (c’) 4000×, (b’, h’) 10,000×, and (d’) 20,000× magnification. Scale bars = 300, 60, 10, 7.5, 3, and 1.5 µm, respectively. EDX analysis confirmed the presence of Ca, P, and O on (a–d, i) random and (e–h, j) aligned mechanically stimulated fibers. EDX images were taken at 4000x magnification and scale bar = 9 µm.

SEM images visualizing cells cultured onto PCL fibers on day 21 are shown in Figure 4. Cells positioned themselves according to the fiber orientation they were cultured on and started to cover the fibrous matrixes. Deposits were observed on random and aligned fiber sheets (Fig. 4(a’–h’)]. In order to assess the composition of those deposits chemical analysis was performed, which confirmed that deposits shown on high magnification images contained calcium (Ca), phosphate (P), and oxygen (O) suggesting mineral deposition [Fig. 4(a–j)].

**Figure 5:**
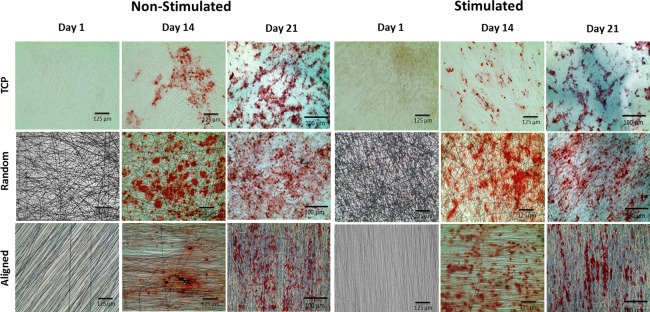
Deposition of calcium onto random fibers, aligned fibers, and TCP. hBMSC were cultured onto PCL fibers and TCP for 21 days and either subjected to hydrostatic pressure or cultured statically. Alizarin red stain visualized calcium deposition onto the tissue culture formats indicating that calcium was deposited throughout the duration of the experiment. Scale bar = 100 µm and 125 µm.

**Figure 6:**
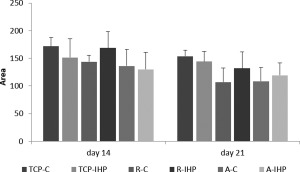
Calcium deposition on TCP and PCL fibers. The alizarin red stained area was quantified for mechanically stimulated (IHP) and non‐stimulated (C) tissue culture formats. Mechanical stimulation resulted in higher calcium content on nanofiber well plates. SD is shown as error bars (*n* = 3).

Alizarin Red stain was performed to detect calcium deposition on TCP, aligned and random oriented fibers on days 1, 14, and 21 of the experiment (Fig. 5). Positive stains were observed on days 14 and 21, but not on day 1 indicating that calcium was deposited throughout the duration of the experiment. Calcium deposition also seemed to be in alignment with fiber orientation. Aligned calcium deposition was observed on aligned fibers and random more evenly spread calcium deposits were seen on random fibers. Representative images used for the semi‐quantitative analyses of alizarin red stains on days 14 and 21 are shown in Figure 5. Alizarin red quantification is shown in Figure 6. For days 14 and 21, analysis of TCP well plates revealed stronger mineralization on non‐mechanically stimulated control plates. A similar trend was observed for aligned fiber well plates on day 14, but random fibers promoted more calcium deposition. On day 21 larger areas of calcium deposition were observed on mechanically stimulated aligned and random PCL fiber sheets compared with non‐stimulated controls. No statistical significant differences were observed comparing the here investigated cell culture formats.

### Gene expression of osteogenic and chondrogenic markers

**Figure 7:**
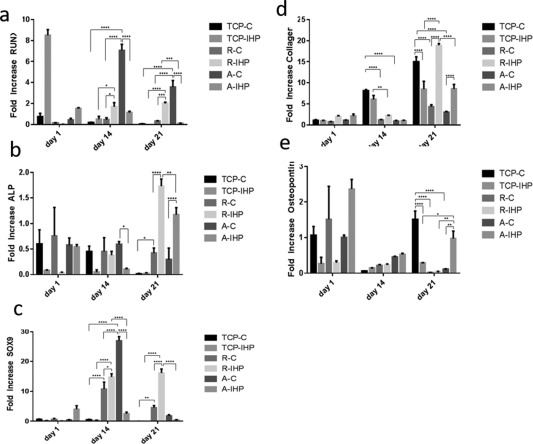
Expression of matrix markers and transcription factors. hMSC were cultured onto random (R) and aligned (A) PCL fibers and TCP, which were subjected to hydrostatic pressure (IHP) and cultured under static conditions (C). To determine expression levels of (a) RUNX2, (b) ALP, (c) SOX9, (d) Col‐I, (e) Osteopontin qPCR was performed. Fold increase relative to GAPDH was plotted over time and SEM is shown as error bars (*n* = 5).

qPCR was performed to investigate expression levels of the osteogenic and chondrogenic markers ALP, RUNX‐2, SOX‐9, OPN, and COL‐I (Fig. 7). mRNA levels for RUNX‐2 decreased for TCP and aligned fibers following mechanical stimulation, but increased for random fibers over the 21 days experiment.

RUNX‐2 expression is significantly increased when comparing R‐IHP and R‐C on day 14 (*p* values = 0.0315) and day 21 (*p* values = 0.0009). For aligned fibers lower levels of RUNX‐2 expression were detected for A‐IHP compared with A‐C on day 14 (*p* values < 0.0001) and day 21 (*p* values < 0.0001). No significant differences were observed for TCP‐C and TCP‐IHP for the duration of the experiment. Runx‐2 expression was significantly higher for A‐C compared with TCP‐C and R‐C for both day 14 (*p* values <0.0001) and day 21 (*p* values = 0.0001). Mechanically stimulated samples were compared with each other, which resulted in higher RUNX‐2 levels for R‐IHP compared with TCP‐IHP on day 14 (*p* values = 0.0429) and day 21 (*p* values < 0.0001) as well as R‐IHP compared with A‐IHP on day 21 (*p* values < 0.0001).

Overall, lower levels of ALP mRNA were detected on control samples for all tissue culture formats (Figure 7b), with the exception of A‐C compared with A‐IHP on day 14, where hydrostatic pressure resulted in significant downregulation of ALP (*p* values = 0.0253). No significant differences in ALP expression were observed for TCP‐C and TCP‐IHP on days 14 and 21. After 21 days ALP is expressed at significant higher levels R‐IHP compared with R‐C (*p* values < 0.0001). A similar trend was observed when comparing A‐IHP with A‐C (*p* values < 0.0001). Overall, highest ALP levels were detected for R‐IHP compared with TCP‐IHP (*p* values < 0.0001) and A‐IHP (*p* values = 0.0073). Furthermore, fiber alignment (A‐IHP) resulted in significant up‐regulation of ALP compared with TCP (TCP‐IHP, *p* values < 0.0001).

Well plates cultured under static conditions revealed an initial up‐regulation for SOX‐9 on random and aligned fibers (day 14 compared with day 1) followed by decreased expression on day 21 [Fig. 7(c)]. SOX‐9 expression decreased for TCP over 21 days and was not statistically different between TCP‐C and TCP‐IHP. When comparing non‐stimulated control samples highest SOX‐9 levels were detected for all PCL fiber substrates compared with TCP on day 14, namely R‐C versus TCP‐C (*p* values < 0.0001) and A‐C versus TCP‐C (*p* values < 0.0001) as well as R‐C versus TCP‐C on day 21 (*p* values = 0.0035).

Hydrostatic pressure further resulted in higher expression of SOX‐9 on random fibers and down‐regulation on aligned fibers and TCP over 21 days. Higher SOX‐9 expression was observed for R‐IHP versus R‐C on day 14 (*p* values = 0.0127) and day 21 (*p* values < 0.0001). Whereas on day 14 cells cultured on A‐C expressed significantly higher level of SOX9 (*p* values < 0.0001) compared with A‐IHP. SOX‐9 expression is then downregulated on A‐C and insignificantly different from A‐IHP on day 21. In addition, qPCR analysis revealed significantly higher SOX‐9 levels for R‐IHP compared with TCP‐IHP and A‐IHP on days 14 and 21 (*p*‐values < 0.0001).

Col‐I expression was upregulated for all tissue culture formats over the duration of the experiment [Fig. 7(d)]. When samples were cultured under static conditions, higher collagen type‐I levels were detected for TCP‐C compared with R‐C and A‐C (*p*‐values < 0.0001) for days 14 and 21 and no significant differences were observed between random and aligned fibers (R‐C vs. A‐C). TCP‐IHP revealed higher COL‐I expression compared with R‐IHP (*p* values = 0.0011) and A‐IHP (*p* values < 0.0001) on day 14. However, after 21 days the expression levels for the matrix marker COL‐I are significantly higher for R‐IHP versus R‐C and A‐IHP versus A‐C (*p*‐values < 0.0001). However, for standard TCP the control samples T‐C showed significantly higher expression of COL‐I than their mechanically stimulated counterparts TCP‐IHP (*p* values = 0.0001). Furthermore, COL‐I expression on R‐IHP is significantly increased compared with A‐IHP and TCP‐IHP (*p*‐values = 0.0001). These results suggest that more collagen was deposited by hBMSC when cultured on mechanically stimulated random fibers.

OPN, an abundant protein in bone is higher expressed for mechanical stimulated fibers R‐IHP and A‐IHP as well as TCP‐IHP on day 14 with highest OPN mRNA levels detected on A‐IHP. However, no significant differences were observed for all sample groups. After 21 days, OPN is significantly upregulated on TCP‐C compared with R‐C and A‐C (*p*‐values < 0.0001). Furthermore, TCP‐C exhibited significantly increased OPN expression compared with TCP‐IHP (*p* values = 0.0001), but no significant differences were observed between R‐C and R‐IHP. Mechanical stimulation on A‐IHP resulted in significant upregulation of OPN compared with A‐C (*p* values = 0.0031). Among all sample groups cells grown on A‐IHP expressed the highest OPN levels compared with TCP‐IHP (*p* values = 0.0320) and R‐IHP (*p* values = 0.0013) for day 21.

These data indicate that the osteogenic potential of hBMSC cultured on non‐stimulated cell culture substrates is lower compared with stimulated fibers on day 21. Changes in gene expression levels suggest that hydrostatic pressure had a more distinct osteogenic effect on cells cultured on random fibers compared with aligned fibers. Hydrostatic pressure resulted in the upregulation of COL‐1 and ALP for aligned and random fibers compared with TCP. This observation shows the relationship between cell and fiber morphology in combination with mechanical stimulation.

## DISCUSSION

This study aimed to provide a better understanding of how mechanical stimulation in synergy with PCL fiber alignment affect the osteogenic potential of hBMSC and hence their orthopedic regenerative potential. To the author's knowledge, the combinatory effect of topography and hydrostatic pressure on hBMSC for bone tissue engineering has not been studied previously.

For tissue regeneration it is essential to design and manufacture artificial matrixes, which resemble the structure and functionality of the native extracellular matrix (ECM).^28^, ^29^ ECM is a dynamic 3D microenvironment, where signals are transmitted between cells and ECM, which enables cell communication, adhesion, proliferation and differentiation. Bone matrix forms a nanometer‐sized composite of organic and inorganic materials with proteins in the range of 50–500 nm.^30^, ^31^ Electrospinning has been widely used to fabricate nanoscale substrates.^10^, ^11^, ^12^, ^17^, ^18^, ^22^, ^28^ Here, hBMSC orientation and morphology followed the orientation of aligned and random PCL nanofibers. MSC on aligned fibers were elongated and spindle‐like in shape due to an extension of the cytoskeleton, whereas cells on random fibers appeared rounded. Similar observations were described by other authors, where random‐oriented and aligned nanofibers had been shown to direct alignment and elongation of several cell types.^17^, ^18^, ^32^, ^33^, ^34^ Another important aspect of controlling cell fate and tissue regeneration are chemotaxis and chemokinesis. Chemotaxis is the directed movement of cells due to external chemical stimuli, so called chemoattractants, whereas chemokinesis, the random movement of cells.^35^ Cell migration can be influenced by a variety of factors including chemical, mechanical, and topographic cues. Traditionally, chemical cues have been utilized to direct cell migration.^36^ Therefore, chemoattractant gradients have been incorporated into electrospun nanofibers to facilitate cell migration and promote tissue formation.^36^ More recently, fiber alignment alone, but also in combination with chemical cues, was shown to direct cell migration.^36^ The migration of hMSC has been widely studied in 2D, but stem cell migration in 3D remains hugely elusive.^35^ Future studies will investigate whether the synergistic effect of chemical, topographical and mechanical cues influences hMSC migration and hence tissue regeneration.

Cells were viable on all culture formats when either subjected to hydrostatic pressure or cultured under static conditions. Higher cell metabolic activities were observed for random fibers. Significant differences in the viability of cells cultured on random and aligned fibers, with random fibers demonstrating a better cellular compatibility than aligned fibers or TCP.^37^ This is assumed to be due to random fibers mimicking the structure of the native bone ECM more closely than aligned fibers.^17^, ^18^, ^22^ In addition, higher metabolic actives were observed for all cell culture formats subjected to mechanical stimulation, suggesting that IHP enhanced cell proliferation. This observation is in agreement with literature.^38^, ^39^ Hydrostatic pressure was found to enhance the proliferation of BMSC through the activation of the cell cycle and further studies have shown that hydrostatic pressure‐promoted cell cycle initiation greatly influenced by RhoA and Rac1 signaling.^38^, ^39^ SEM, EDX analysis, and positive alizarin red stain indicated calcium deposition on aligned and random fibers as well as TCP in static and dynamic culture. Calcium was deposited according to cell orientation on PCL fibers. This observation is in agreement with previously published studies where random and aligned PCL nanofibers have been shown to promote osteogenesis.^10^, ^11^, ^12^, ^22^ Furthermore, MSC cultured on TCP have been shown to undergo osteogenic differentiation when cultured in osteogenic medium.^40^ Hydrostatic pressure seems to result in more condense, larger calcified nodules.

It is assumed that fiber orientation and mechanical stimulation are key factors influencing cell fate in this study. Mechanical stimulation using hydrostatic pressure altered the mRNA levels for the investigated osteogenic, chondrogenic and matrix markers indicating a clear effect on cell differentiation. Clear trends in the expression patterns of RUNX‐2, ALP, SOX9, Col‐I, and OPN were described for the investigated tissue culture formats. Expression of osteo‐chondrogenic and matrix markers were significantly increased in response to hydrostatic pressure in cells grown on random fibers compared with aligned fibers and TCP. Results further suggested that mechanical stimulation had a more pronounced osteogenic effect on cells grown on random fibers over aligned fibers, which demonstrates the relationship between fiber morphology, cell shape, and mechanotransduction. Cells adhered along the nanofibers exhibit a stretched morphology. This could be of great importance as cytoskeletal rearrangements and tension, and hence cell shape has been demonstrated to affect gene expression.^41^, ^42^, ^43^ Integrin and the cytoskeleton are crucial for stem cell behavior and for converting mechanical cues into intracellular signals.^44^, ^45^, ^46^ It has been hypothesized that receptors are stretched and activated when cells stretch themselves resulting in changes in gene expression.^41^ Consequently, random and aligned nanofibers influence stem cell differentiation and gene expression by affecting the cell morphology.^43^ This could account for the differences in gene expression observed between non‐stimulated random and aligned fibers.^43^ Moreover, differences between TCP and nanofibers could result from fibrous cell culture substrates resembling the native ECM more closely than standard tissue culture formats. Therefore, MSC gene expression is influenced by the surface topography (fibrous vs. flat).^4^ Kolambkar et al. (2014)^47^ revealed that fiber alignment had a significant effect on ALP activity. Higher degree of alignment resulted in decreased ALP activity as has also been shown in this study. Since MSC express only low levels of ALP its production is a critical biochemical indicator for the presence of osteoblasts.^34^ Increase in ALP expression levels confirms the differentiation of MSC into osteoblasts and their subsequent mineralization.^34^ Here, more calcium was deposited on random fibers meshes. Alignment did not enhance calcium deposition, possibly because cells are more migratory on aligned fibers.^47^

Random PLA fiber meshes were found superior compared with aligned fibers in terms of osteogenic potential.^48^ The extracellular signal‐regulated kinase‐mitogen‐activated protein kinase pathway is important for the expression of RUNX2 initiating osteogenic differentiation of MSC. ECM shape influences the MSC actin cytoskeleton through integrins and hence cell differentiation.^48^ RUNX‐2 is known as the principle transcription factor for osteoblast differentiation. It is expressed in MSCs at the onset of skeletal development and is present in osteoblasts throughout their differentiation.^49^ Highest RUNX‐2 expression was detected for non‐stimulated aligned fibers on days 14 and 21. RUNX‐2 was non‐significantly downregulated on day 21 compared with day 14 and this is in agreement with previously published data.^21^ In the case of A‐IHP RUNX‐2 expression was non‐significantly different indicating that hydrostatic pressure did not cause the downregulation. These results indicate that in static culture aligned fibers seem to favor osteoblastic differentiation of hBMSC. At later time points RUNX‐2 levels however decrease. Mechanical stimulation of random fibers on the other hand seems to have a positive effect on osteoblast development since RUNX‐2 levels are more highly expressed (days 14 and 21). Hydrostatic pressure resulted in RUNX‐2 upregulation on days 14 and 21. R‐IHP compared between days 1 and 14 was significantly increased (*p* values = 0.092) and day 1 compared with day 21 (*p* values = 0.0006).

The transcription factor SOX‐9 follows similar expression levels as RUNX2. SOX‐9 is critical for chondrocyte differentiation and function. It is an indicator for the commitment of osteochondroprogenitor cells and hence endochondral or intramembranous ossification, chondrogenic mesenchymal condensation as well as chondrocyte proliferation, differentiation, and maturation.^50^ ALP expression was higher on random stimulated and non‐stimulated fibers. Mechanical stimulation of random fibers resulted in higher COL‐1 expression and OPN expression was increased on mechanical stimulated aligned fibers compared with other cell culture formats on day 21. COL‐1 is the major protein in extracellular matrix of connective tissues. It is the main protein in bone (95% of collagen in bone is collagen type‐I) and is considered an indicator for bone matrix deposition.^51^ OPN is an abundant protein in bone, localized to cell–matrix and matrix–matrix interfaces. It promotes attachment of bone cells to bone matrix. Research has shown that IHP resulted in increased OPN and ALP expression in osteoblasts and osteoblast‐like cells.^52^, ^53^, ^54^

Expression patterns during osteogenic differentiation of stem cells have been divided into a proliferative phase, a matrix deposition stage and a mineralization phase. OPN is expressed with the beginning of the mineralizing phase, which is an essential marker for mineralization.^34^ Regarding this differentiation pattern and the results obtained in this study, matrix deposition and fiber mineralization was confirmed. It was previously hypothesized that hydrostatic pressure caused mineralization through exerting physical force onto hBMSC‐seeded artificial matrixes.^25^ Another possible explanation could be changes in physiological parameters such as oxygen, carbon dioxide and pH of the cell culture medium.^55^ The exposure of cell‐seeded constructs to hydrostatic pressure resulted in increased dissolved oxygen and carbon dioxide concentrations as well as a decreased pH of the surrounding medium as shown in a previous study published by our group.^25^ When cells are subjected to alterations in their biophysical environment, changes in physiological parameters are known to influence mineralization and cell activity.^56^ This study investigated the influence of random versus aligned fibers on their own (statically cultured control samples), but also the synergistic effect of hydrostatic pressure and fiber alignment (mechanically stimulated samples). For both sample groups we see an increase in mineralization, suggesting that the fiber substrates on their own already have a positive effect on mineralization. Fiber alignment in combination with hydrostatic pressure resulted in a further increase in mineralization. Generally, random fibers seemed more suitable for early stage osteogenesis of MSC *in vitro*. A clear difference was observed between aligned fibers and random fibers in mineral deposition. Aligned and random fibers used in this study were different in fiber diameter. Random PCL fibers exhibited slightly smaller average fiber diameters compared with aligned fibers, but had a broader diameter size range. The random fiber diameter was similar to the range of ECM proteins (50–500 nm). These differences in fiber diameter might also have contributed to the observed cellular behavior. Lyu et al. (2013)^21^ observed negligible effects on MSC differentiation for fiber diameters in a range between 300 and 1200 nm. However, the comparison of micro‐and nanometer‐sized fibers indicated that cell viability and differentiation were greatly enhanced on nano‐scaled fibers assumingly because their resemblance to native ECM. Yoshimoto et al. (2003)^10^ and Fujihara et al. (2004)^11^ assessed the osteogenic potential of bone‐like cells seeded onto PCL nanofibers with diameters of 400, 760, and 900 nm. Results indicated that all fiber meshes were suitable substrates for cell adhesion, matrix, and calcium deposition.^1^ The comparison of nanofibers (diameter ∼440 nm) and microfibers (diameter ∼4.3 µm) for inducing chondrogenic differentiation of MSC revealed higher mRNA levels of chondrogenic markers, cell proliferation and matrix production for micrometer‐sized fibers.^57^ However, the comparison of fiber diameter on stem cell fate across various studies is difficult due to utilization of different polymer materials and cell types.^58^ Even though, aligned and random fibers used in this study exhibited differences in fiber diameters, it is not assumed that these differences are responsible for the observed trends in cell viability and metabolic activity as well as gene expression.^47^ In order to separate the effect of fiber diameter and fiber orientation, random and aligned fibers of the same fiber diameter need to be investigated. Further investigation is required to examine the influence of different fiber diameters in combination with mechanical stimulation and biochemical cues and to design the optimal culture conditions for successful bone regeneration. In addition, the production of fiber meshes with sufficient thickness and pore size to promote cell penetration and migration into the 3D matrixes as well as donor variability will be investigated in future studies.

## CONCLUSION

This study aimed to investigate the influence of topography and hydrostatic pressure on stem cell differentiation for the regeneration of bone tissue. Our results demonstrated that cells will align according to fiber orientation, which has been previously described, but further established that the effects of IHP are more pronounced on random fiber substrates. Cells on aligned substrates, in contrast, are less responsive to the intermittent pressure regimes. This is of great importance for the design of multiple therapeutic regenerative medicine strategies for bone tissue engineering.
